# The influence of intraseasonal oscillations on rainfall variability over Central Africa: case of the 25–70 days variability

**DOI:** 10.1038/s41598-023-46346-y

**Published:** 2023-11-13

**Authors:** Claudin Wamba Tchinda, Alain Tchakoutio Sandjon, Angennes Lucie Djiotang Tchotchou, Audryck Nzeudeu Siwe, Derbetini A. Vondou, Armand Nzeukou

**Affiliations:** 1 Laboratory for Environmental Modelling and Atmospheric Physics, Department of Physics, Faculty of Science, University of Yaoundé I, Yaoundé, Cameroon; 2 Department of Computer Science Including Basic Sciences, Higher Technical Teacher’s Training College Kumba, University of Buea, Buea Road, P.O Box 249, Kumba, Cameroon; 3 Laboratory of Industrial Systems and Environmental Engineering, Fotso Victor University Institute of Technology, University of Dschang, Bandjoun, Cameroon

**Keywords:** Climate sciences, Environmental sciences

## Abstract

The influence of intra-seasonal oscillations (ISO) on rainfall in Central Africa (CA) during the March–May (MAM) season is assessed using the National Oceanic and Atmospheric Administration Climate Prediction Center daily gridded rainfall data. ISO indices are defined using the time series analysis of the first two principal components resulting from the empirical orthogonal function, applied to daily filtered outgoing longwave radiation. Based on these indices, a total of 71 strong Intraseasonal Events (SIEs) and 66 weak Intraseasonal Events (WIEs) were selected using threshold method. The results show that SIEs are associated with enhanced rainfall conditions over almost all the study area, while WIEs provide a meridional dipole-like rainfall pattern, consisting of increasing precipitation in the western part and decreasing in the eastern part of CA. The relationship with Madden–Julian Oscillation (MJO) was also examined. The positive rainfall anomalies associated with MJO phases progress eastward and are modulated by the 850 and 200 hPa horizontal wind. The circulation, linked to geopotential height anomalies at lower layers, tends to strengthen (reduce) the convective activity over the region during extreme ISO events and for the MAM season throughout the study period. Uncentered pattern correlation was further used to assess the link between ISO and MJO phases during the MAM season and we found a correlation of 0.5 in precipitation anomalies between phases 1 and 2 of the MJO and the SIEs; − 0.4 and − 0.6 between phases 5 and 6 and the SIEs respectively, suggesting a strong relationship between ISO events and MJO.

## Introduction

Rainfall in the tropics is one of the most difficult atmospheric parameters to predict because of the complexity of the processes involved in its generation. Meanwhile, the economy of Central Africa (CA) countries strongly depends on rain-fed agriculture, hydro-power generation and forest products. Therefore, the understanding of the variabilities of rainfall within a season is critically needed for socio-economic activity planning. The area of this study extends from 0°–50°E to 15°S to 15°N mostly over the continent and part of the Indian and Atlantic Oceans. The topography of the region highly varies from one location to another. Western part consists of plains and Mount Cameroon, the eastern part consists of highlands, plateaus and rifts. In addition, improving our knowledge of the mechanisms driving intra-seasonal events can be of great importance to improve seasonal to subseasonal weather forecasting over the tropics. This is even as important as in recent decades, many African regions have been affected by rainfall variability and long-term changes in rainfall distribution and intensity. Generally defined as fluctuations with periods longer than the synoptic scale but shorter than the seasonal time scale, the ISO are a prominent feature of tropical weather, particularly in the Indo-Pacific region^1–3^. The ISO was initially identified by Madden and Julian^1^ where they found a peak in the co-spectrum of the 850 and 150 mb zonal wind components in the period range 41–53 days. Since then, many researchers greatly documented the ISO patterns in different regions around the tropics (e.g.:^4–18^). Tazalika and Jury^19^ investigated the spatial distribution of ISO over Central Africa and found three dominant modes of variability. Few years later, Sandjon et al.^20^ studied the ISO patterns in CA and confirmed that the three dominant modes of variability exhibit high spatial loadings over Northern Congo, Southern Ethiopia, and Southwestern Tanzania, respectively. Moreover they showed that the ISO intensity globally decreases from one decade to another between 1980 and 2010.

The intraseasonal rainfall variability in CA has two dominant frequency bands: the 10–25-day band and the 25–70-day band. This variability has been studied extensively, enriching our understanding of the region's climate. For example, Sandjon et al.^21^ analyzed the 10–25-day intraseasonal variability of rainfall over CA, and found that it is more pronounced in eastern than western Central Africa. Zhou et al.^22^ assessed the dominant intraseasonal precipitation mode during the February-May rainy season in Rwanda, finding that it exhibits significant variability on the 10–25-day time scale and is associated with anomalous westerly winds. However, the focus of this study is the 25–70-day frequency band during the MAM season. This is because previous studies by Sandjon et al.^14,23^ and Tchakoutio et al. (2021) have shown that the intensity of the 25–70-day mode peaks during the MAM season. The MAM season is a typical rainy season in Central Africa and has been the subject of several studies^24–26^.

Although the aforementioned studies investigated the impact of ISO on the intra-seasonal variability of precipitation across Central Africa, they mostly focused on the ISO patterns and total rainfall distribution, and did not really investigate the mechanisms of ISO impact on precipitation. No particular attention was carried out on the MAM season and on how the MJO modulates the ISO as well as the response of the coupling MJO-ISO on the rainfall system. Yet, understanding such a coupling is critically needed to improve seasonal to subseasonal forecasts, which are strongly required for agricultural and other socio-economic activity planning.

The aim of this study is therefore to examine the impact of intraseasonal oscillation 25–70-day on rainfall variability over CA during the MAM season. The link between the MJO indices and ISO is also assessed. The response of the region’s atmospheric circulation features such as convection, the upper and lower layers’ atmospheric circulation are also investigated in order to understand the reasons behind the pattern of the rainfall climatology during the MJO-ISO interaction.

The remainder of the paper is structured as follows: the details of the data used and their sources are described in Section "Data and method". The methods employed to extract the signal of MJO and ISO are presented in Section "Results and discussion". Section "Summary and conclusion" discusses the results and the paper ends with a summary and conclusions in Sect. 5.

## Data and method

### Data

#### Outgoing longwave radiation (OLR)

In this study, the daily OLR data is used as a proxy for convection. It is well known that in the tropics, deep convection and rainfall can be estimated through low OLR values (values less than a well-chosen threshold). It has been successful to highlight the evolution of convection over tropical Africa^27^, and over many other regional regions (e.g.^28–34^). The OLR is spatialized on a 2.5° × 2.5° grid^36^ and covers the period from 1980 to 2019, with gaps filled by temporal and spatial interpolation. The OLR datasets are available on the NOAA website http://www.esrl.noaa.gov and can be freely downloaded.

#### Climate prediction center (CPC) rainfall data

Daily rainfall data were used to establish the degree of association with MJO. This data set from the Climate Prediction Center (CPC) Unified (Chen et al.^36^) over Central Africa, available on a 0.5° × 0.5° fixed latitude–longitude grid, is based on station precipitation observations. CPC-Unified draws on archives of station observations, including Global Historical Climate Network, the Global Summary of the Day, the World Meteorological Organization’s Global Telecommunication System, and national and international agencies. CPC-Unified considers orography and implements quality control techniques based on comparisons with satellite-derived precipitation and model forecasts. It has been used in many studies to evaluate rainfall over the world (e.g.: Thorne et al.^37–41^. CPC Global Unified Precipitation data provided by the NOAA/OAR/ESRL PSD, Boulder, Colorado, USA, from their Web site at https://www.esrl.noaa.gov/psd/

#### NCEP–NCAR reanalysis winds and geopotential heights

Global analyses of winds and geopotential heights are obtained from the National Centers for Environmental Prediction–National Center for Atmospheric Research (NCEP–NCAR) reanalysis^42^ on a 2.5 grid. Using data from 1980 to 2019, we maximize the benefits of the input of satellite observations into the reanalysis. We are thus confident in the representation of the circulation that it provides. The daily Zonal and meridional wind fields are deemed to be “most trustworthy” due to their direct dependence on instrumental measurements^11,12^. Datasets are available at 2.5° × 2.5° latitude–longitude resolution. The indices for MJO used in this study are Real-time Multivariate MJO (RMM) Indices defined by Wheeler and Hendon^43^, for the period of 1980–2019. These indices are available for download on the following web site: https://www.psl.noaa.gov/mjo/mjoindex/rMII_index_latest.txt. To extract MJO components from the 850 hPa, 200 hPa zonal wind and OLR, the annual cycle and components of interannual variability are removed. Two principal component time series from multivariate EOF of the MJO components are defined as RMM1 and RMM2. These scores are normalized by their standard deviations over the studied period.

### Methodology

#### Lanczos filtering

In atmospheric science, digital filtering is often used to isolate frequencies that are of interest in a dataset. Lanczos filtering is a technique that has been widely used to to extract desired timescale in the atmospheric fields around the tropics^14,21,23,44,45,46^. This filtering technique involves the use of a specific type of window function known as the Lanczos window, defined as:

1 $$w\left(n\right)=sinc\left(\frac{2n}{M-1}-1\right)$$

where $sinc\left(x\right)=\frac{sin\left(\pi x\right)}{\pi x}$ and ‘M’ is the number of points in the output window.

This window function has proved particularly effective for climate analyses, offering a balance between reduced Gibbs oscillations and good properties in spectral domains^47,48^.

#### Empirical orthogonal function (EOF) analysis

EOF analysis is a statistical technique that reduces the dimensionality of a dataset while retaining as much information as possible. Over the years, the EOF analysis technique has been widely used in meteorology and climatology particularly for understanding precipitation variability by identifying the major mode of variation^43,45,49–51^. It is a multivariate process that transforms correlated data into uncorrelated variables, hence lowering variability. The first principal component defines the most variation, while the second explains the most variation^52^. The EOF analysis is based on the following mathematical formulas:

The covariance matrix:

2 $$M=cov\left(X\right)$$

where ‘M’ is the covariance matrix, ‘X’ the data matrix and ‘cov()’ is the covariance function;

The eigenvectors and eigenvalues of the covariance matrix:

3 $$\left(M-\lambda I\right)v=0$$

where ‘v’ is the eigenvector, ‘$\lambda$’ is the eigenvalue and ‘I’ is the identity matrix;

Projection of the data onto the principal component:

4 $$x^{\prime}=X*v$$

where ‘$x^{\prime}$’ is the projection of the data on to the principal component, ‘X’ the data matrix and ‘v’ is the eigenvector.

Principal components seek to explain the most variance possible using the fewest possible components.

#### ISO indices and selection of sISOs et wISOs

**Figure 1:**
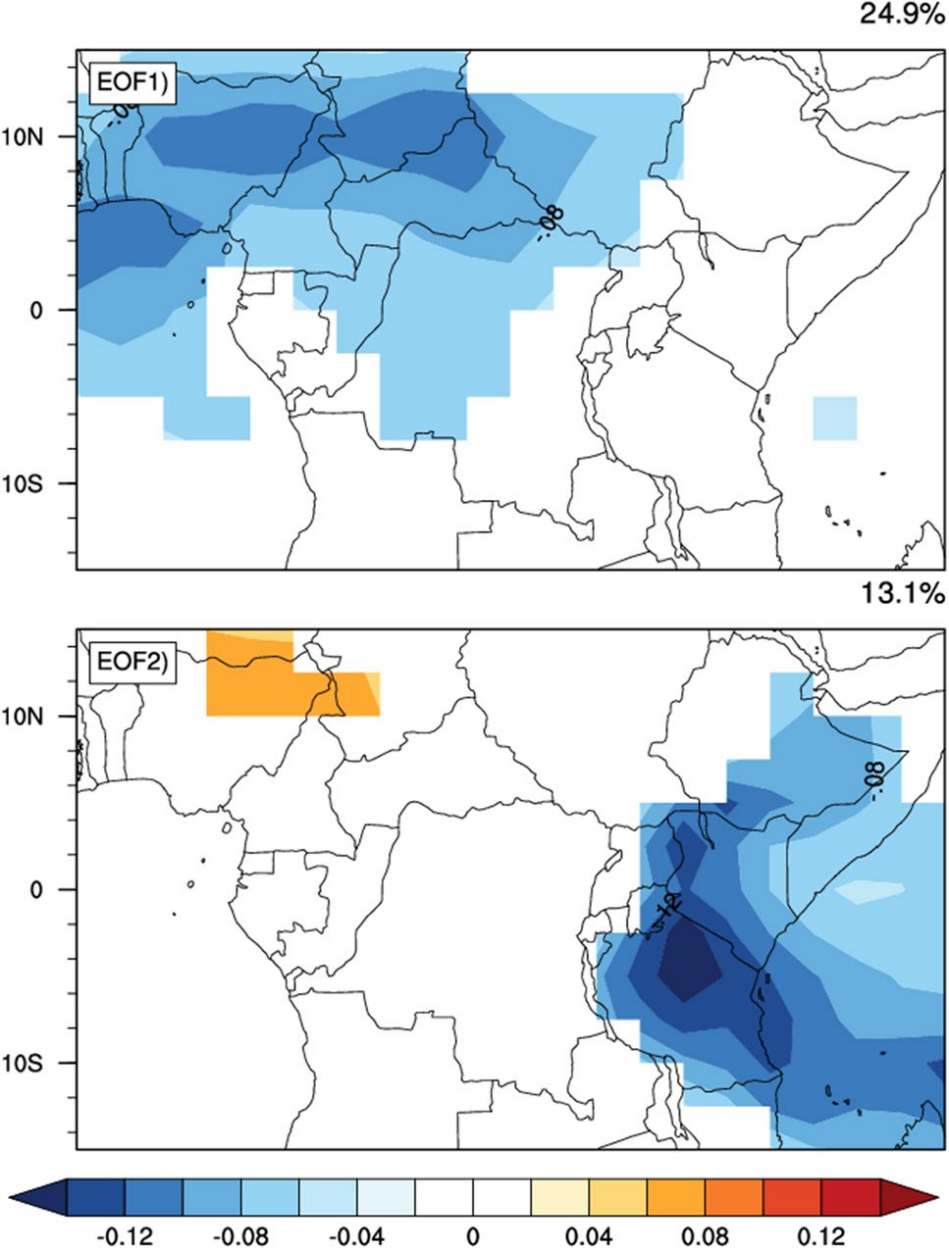
Spatial loadings for the first two EOFs of long time daily filtered OLR anomaly (W/m^2^) over CA, during MAM season and from 1980 to 2019. Values between − 0.05 and 0.05 are not plotted for a better presentation. The corresponding EOF is indicated at the upper left angle in each plot.

We defined strong and weak ISO using the ISO indices computed from the scores of the first EOFs obtained after the EOF analysis on the filtered OLR anomalies. Therefore to extract the intra-seasonal time scales, we first filtered these anomalies using a 25–70-day Lanczos bandpass filter^47^ with 141 weights over the region (15°S–150°N,0–50°E). We then applied a 5 days running mean on the filtered anomalies to remove synoptic-scales signals. The empirical orthogonal functions (EOFs) were calculated from the 25–70-day filtered OLR anomaly as presented in the Fig. 1, for illustration.

The time series of the first two principal components (PC1 and PC2) are then combined to calculate a single vector that measures the overall variation of the ISO amplitude. This vector can represent the ISO intensity and it is expressed as follows:

5 $$A=\sqrt{PC{1}^{2}+PC{2}^{2}}$$

**Figure 2:**
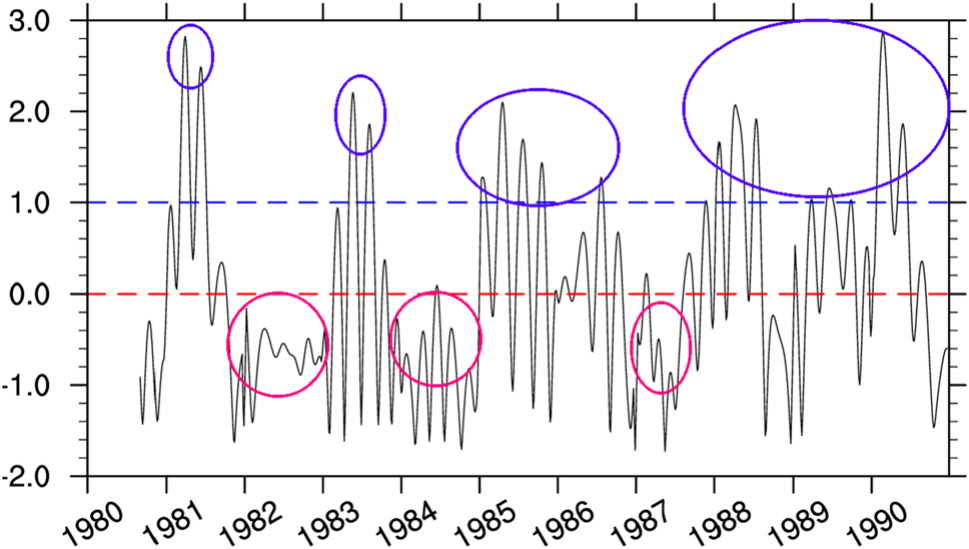
Time series of standardized anomalies of the ISO amplitude, from 1980 to 1990. We presented the results for just 10 years, for a better presentation. The dashed lines represent the references for the selection of IEs. Red is the reference for peaks anomalies greater than 1 and blue is the reference for peaks less than 0.

Moreover it is well known that in meteorology, when seasonal variations are present within a dataset, it often helps to express the data in terms of standardized anomalies. Standardized anomalies are calculated by dividing anomalies (deviation from the mean) by the climatological standard deviation. The standardized data so obtained generally provide more information about the magnitude of the anomalies because influences of the dispersion have been removed. The ISO amplitude index used in this study is the standardized form of the ISO amplitude obtained in Eq. 5

6 $$Anom =\frac{A-\overline{A}}{\sigma }$$

where ‘A’ is the amplitude of the ISO signal, ${\prime}\sigma {\prime}$ is the standard deviation and ‘$\overline{A}$’ is the amplitude mean. ‘*Anom’* is the standardized anomaly denoted now as ISO index. Figure 2 represents the time series of the daily amplitude index of the 25–70-day intraseasonal oscillation over CA from 1980 to 1990. We have plotted this time series over 10 years only, for a better illustration of the different peaks. This figure clearly shows that ISO intensity highly varies with time, with some sequences of extremely high amplitude, followed by periods of relatively low values. Moreover, a deep look into this figure reveals that the ISO intensity is also cyclical, each cycle being characterized by an increase of ISO amplitude that reaches a maximum and then decays subsequently. We then focused on the amplitude peak for each cycle, to define sISOs and wISOs. The sISOs are defined as ISO sequences with standardized amplitude peaks greater than 1 and wISOs, the sequences corresponding to the standardized amplitude peak less than 0. Indeed this method of selection, generally called threshold method, has been widely used by researchers in climate variability studies in many parts of the word, including CA^53,54^, with relevant results. Following this method, one can notice that there are years essentially characterized by sISOs, such as 1981–1982, 1985–1986, and 1988–1990, and some other years mostly characterized by wISOs, such as 1982–1983, 1984–1985, and 1987–1988. The method of extraction of ISO indices here is similar to that used by some authors^43,45^. In fact, after extracting the dominant modes of intra-seasonal variability, these authors have used this method to deduce the ISO index from the first two principal components. Time indexes of these circled peaks were then selected, and are used to build all the ISO composites.

To examine the influence of ISO on the spatial distribution of the intra-seasonal rainfall, a composite of daily filtered rainfall anomalies is presented based on strong and weak ISO events during MAM season over the entire study period. To evaluate the corresponding changes in the circulation and convection, composite of daily filtered 850 hPa and 200 hPa wind, geopotential high at 925 hPa and OLR were also prepared for the two defined ISO events.

In order to assess the influence of MJO on CA rainfall at intra-seasonal time scales, a composite of daily filtered rainfall anomalies is presented based on each phase. Only dates of MAM season during which the amplitude of MJO exceed 1 were considered for the composite.

Composite of Moist static energy, 850 hPa and 200 hPa wind anomaly, geopotential height at 925 hPa were also created to provide additional information on weather conditions. In fact, composite analysis is a powerful technique used to determine some of the basic structural characteristics of a meteorological or climatological phenomenon that are difficult to observe in totality. Many authors have used this technique to explore rainfall distribution during ISO and MJO phases around the world^14,23,51,55–57^.

The relation between the MJO and intra-seasonal rainfall variability was accessed by calculating the uncentered patterns correlation between intraseasonal rainfall at each MJO phase and intraseasonal rainfall during the SIEs/WIEs. This shows the similarity between the two fields^58^.

## Results and discussion

### Structure of the 25–70 days intraseasonal oscillations

**Figure 3:**
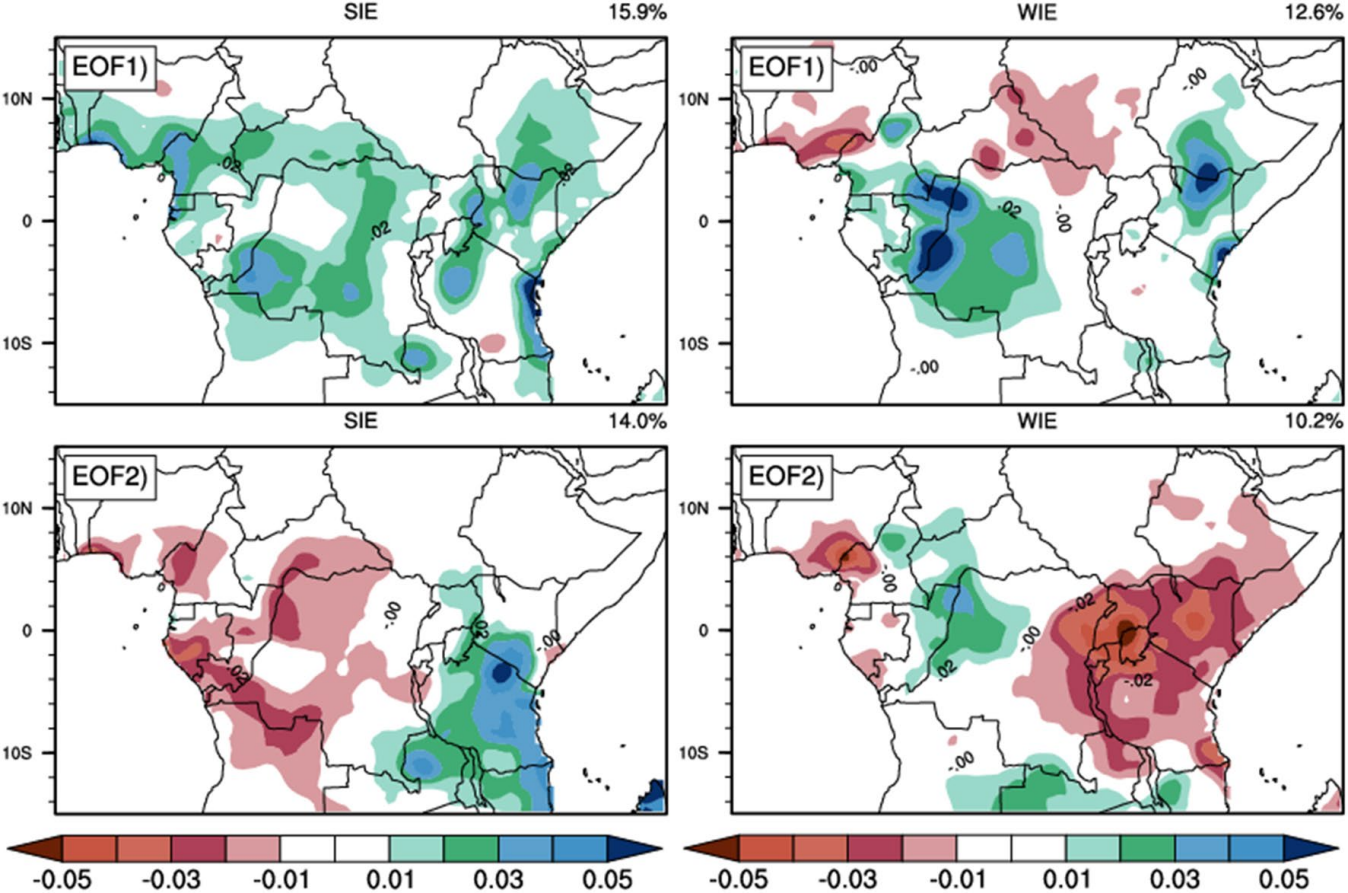
Spatial structure of the 25–70-day filtered daily rainfall anomalies for MAM season associated with the first two principal components during SIEs (left column) and WIEs (right column) and over the 1980–2019 period. The corresponding EOF as well as the percentage of variance explained are indicated at the top left (for EOF) and the top right (for the percentage variance) of each figure.

To investigate the spatial structure of the intraseasonal rainfall variability over CA, we performed EOF analysis on composites of daily filtered precipitation anomalies for the SIEs and WIEs, respectively (Fig. 3). To achieve this, we first calculated the daily anomaly of precipitation before, and then filtered to extract the intraseasonal time scale as described in the methodology section. We therefore created the time series of daily filtered precipitation anomalies. The EOF analysis was performed on the resulting filtered data time series and this was done separately for SIEs and WIEs. The results show that during SIEs, EOF 1 and EOF 2 account for 15.9% and 14% of the total variance, respectively, while during WIEs, they account for 12.6% and 10.2%, respectively. During SIEs, the spatial loadings of EOF1 exhibits a unique pattern that consists of positive loading over most parts of the region, extending within Latitude 10°S to 10°N and Longitude 0° to 50°E over the land. This suggests an increasing trend of rainfall during SIEs in MAM (Djebata et al.^59^). However EOF2 loadings show an East–West dipole pattern that consists of positive loadings over the eastern part extending within Longitude 25°E to 40°E and negative loadings in the western part between 10°S 10°N and Longitude 0° to 25°E. As for WIEs, the EOF1 spatial loadings reveals the overall dominance of positive loadings, with a small area of negative loadings around the latitude of 10°N This feature is almost reversed for EOF2 where positive loadings are found in west and East Africa and around and negative loadings over central Africa.

**Figure 4:**
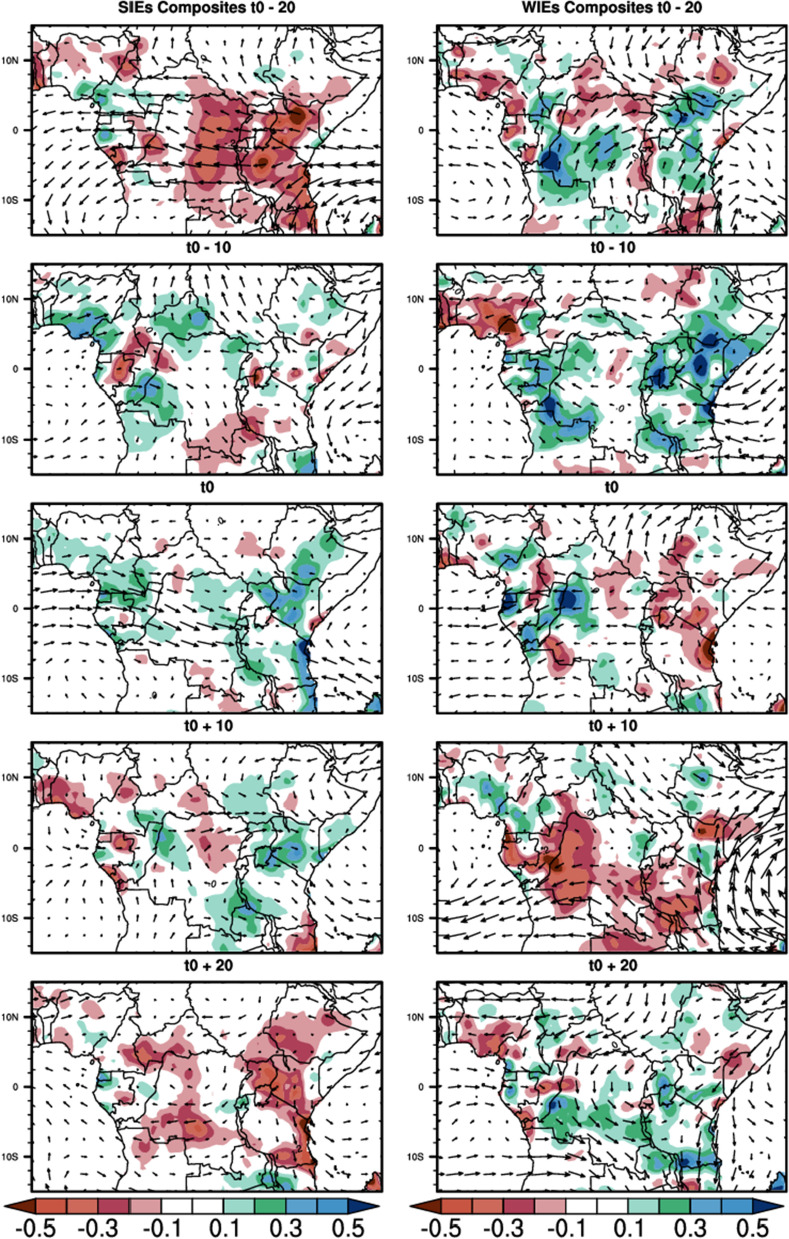
Composites of 25–70-day filtered rainfall anomalies (shaded contours, in mm) and 850 hPa horizontal winds anomalies (in m/s) during SIEs and WIEs for the MAM season. The reference wind vector is 3 m/s.

Figure 4 below shows the time sequence of the composite of filtered rainfall anomalies (shaded) and the 850 hPa horizontal winds anomalies (vectors) from t0-20 to t0 + 20 days in 10-day steps. t0 represent the reference time, that is the time corresponding to the peak intraseasonal events. During SIEs, at the early stage (t0-20), CA is marked by negative rainfall anomalies over almost all the region, except Cameroon and Guinea Atlantic coasts. At the stage t0-10, CA is marked by some spots of positive rainfall anomalies, which grow to reach the mature stage at t0 and extend over most parts of the area. These positive anomalies disappear progressively from the west of the study area, and are replaced by the negative anomaly at t0 + 10, which progressively expands and covers almost the whole area at t0 + 20, as well as at t0-20. For the 850 hPa horizontal winds, during SIEs, we observe at t0-20 that the winds are mostly westward, from Indian ocean to the Atlantic ocean over the Equatorial region (around 2°N–5°S latitude) and southwestward over Atlantic ocean (around 5°S–12°S latitude). At t0-10, northeastward wind anomalies are found over the latitude 5°N–15°N. An anticyclonic circulation dominates over the Indian ocean part of the study area. At t0, the situation is almost opposite to t0-20, the dominant circulation is the Eastward flow around equator (5°S to 5°N latitude) that slopes southeastward from Congo Basin and ends over the East African highlands. Another circulation feature at this stage is the northwestward flow from the Indian ocean that penetrates the continent. This circulation starts to weaken in speed and magnitude at t0 + 10 and the sense in the Indian Ocean is opposite to what it was at t0. At t0 + 20, the circulation is very weak over the continent and is oriented to the Northwest below the Equator in the Atlantic Ocean and to the Southeast in the Indian Ocean. The circulation flow increases during the WIEs and is very scarcely structured, with the development of anticyclone over the Congo Basin and the Indian Ocean. The positive rainfall anomalies are more intense at each stage.

**Figure 5:**
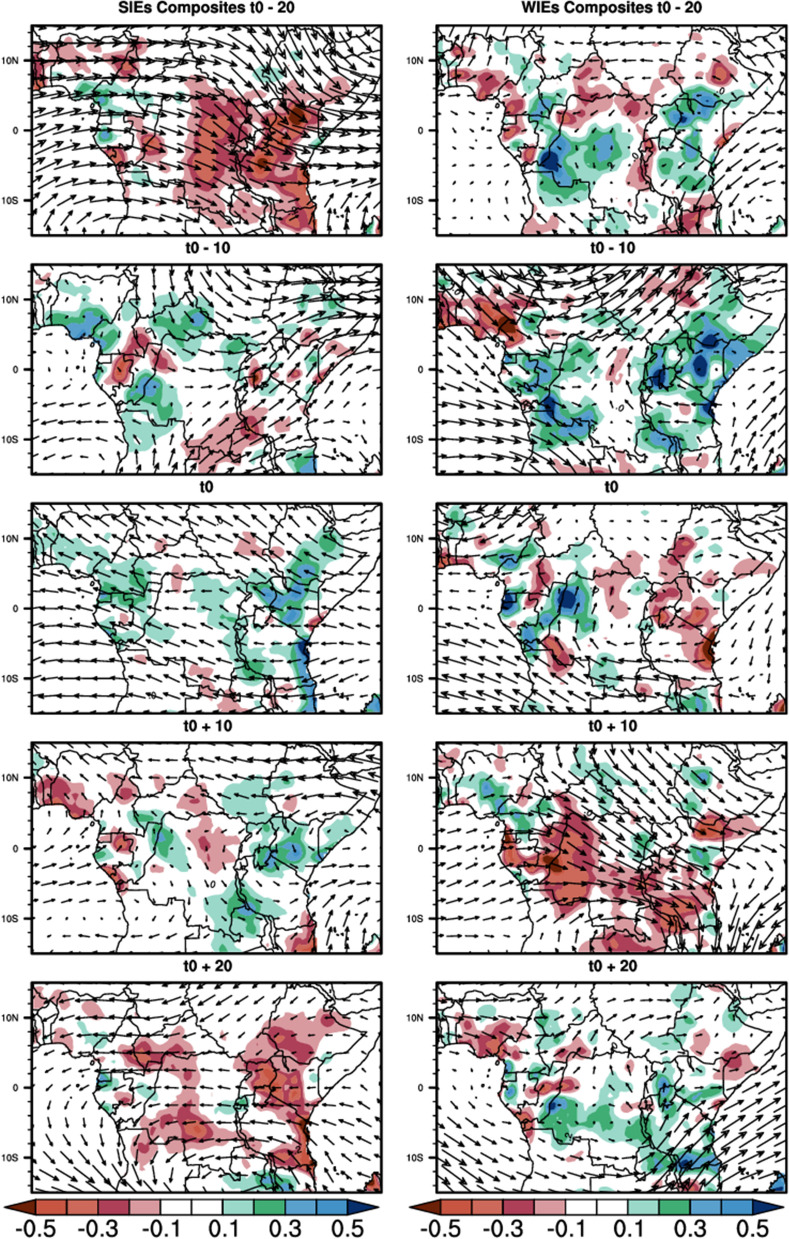
Same as in Fig. 4 but the horizontal wind anomaly is at 200 hPa and the reference vector length is 5 m/s.

At 200 hPa (Fig. 5), the circulation is very intense and varies strongly in direction and sense from one time lag to another. During the SIEs and at t0-20, a wave train crosses the whole study area from west over the Atlantic Ocean to east over the Indian Ocean. This eastward circulation starts to weaken in the west at t0-10, and at t0 it is completely reversed, blowing from east to west over the whole study area. At t0 + 10 the circulation has weakened considerably in the Congo Basin, and swings eastward over the Atlantic Ocean between the Equator and 5°S latitude before stopping over the continental coasts. On the other side of the continent, the wind blows north-eastwards over the Indian Ocean. At t0 + 20 the easterly wind anomalies that cross the continent from the Indian Ocean tilt to the southwest as it exits the continent over the Atlantic Ocean, and returns to the continent below 10°S latitude. During the WIEs, the circulation is weak in the Congo Basin except at t0 + 10 when the wind converges towards the Southeast.

This high variability in the sense, direction and intensity of the circulation, from one time sequence composite to another, at the surface and at high altitude, is associated with the variability in the spatial extent of positive and negative rainfall anomalies in Central Africa, during SIEs and WIEs.

### Influence of ISO peaks on rainfall variability and associated circulation at intra-seasonal time scale

For the entire study period (1980–2019) and for MAM season, we identified 71 SIEs and 66 WIEs.

**Figure 6:**
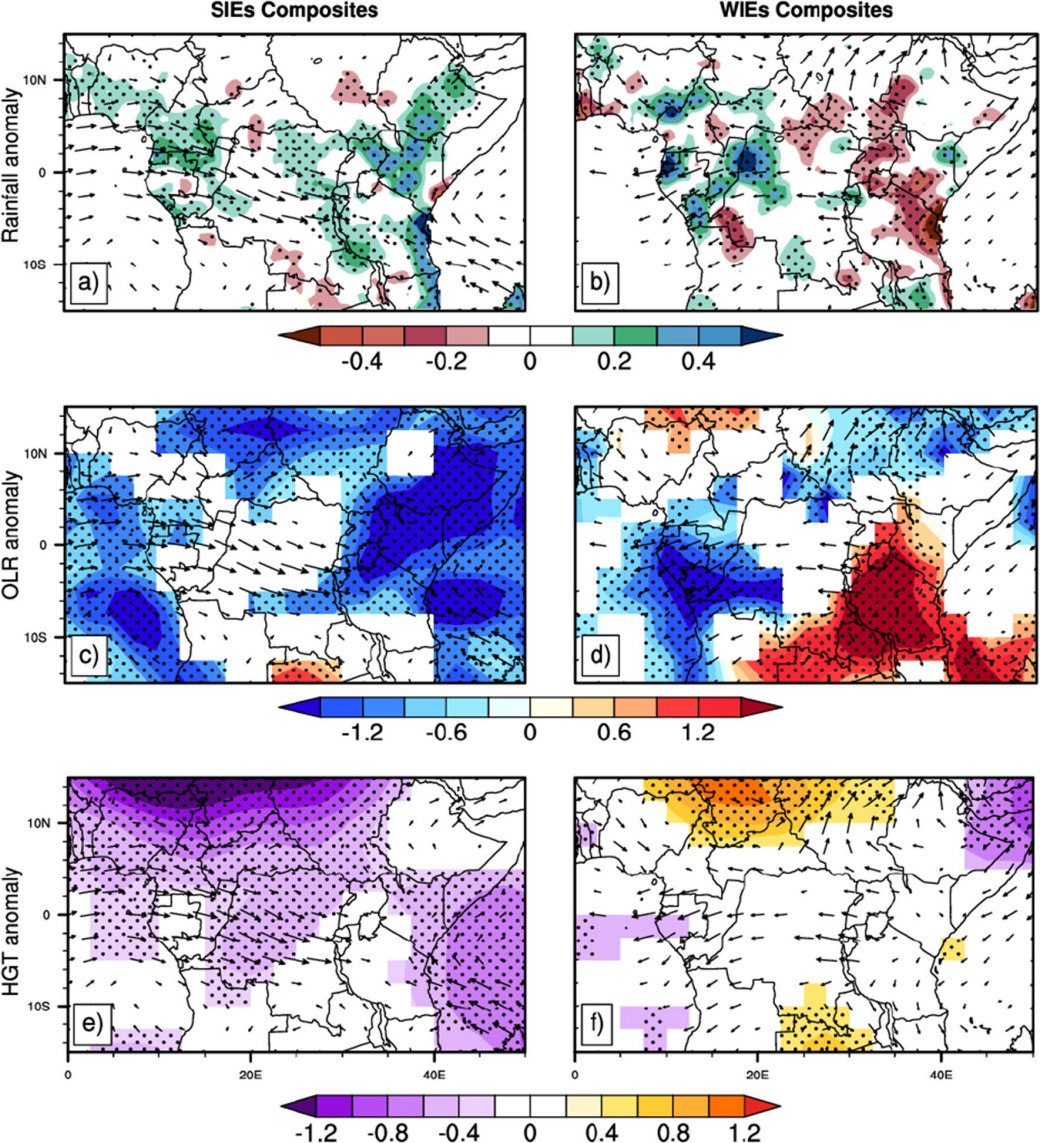
Composite of filtered rainfall (**a**), OLR (**b**) and the geopotential height at 925 hPa (**c**) anomalies, based on SIEs (**a**, **c** and **e**) and WIEs (**b**, **d** and **f**) during MAM season. Dots indicate areas with significant values at 95% confidence level. Arrows indicate filtered wind anomalies at 850 hPa. Anomalies between − 0.01 and 0.01 m/s are not represented.

Figure 6 highlights, for the 71 SIEs, the spatial distribution of daily filtered rainfall anomalies (a), daily filtered OLR anomalies (c), daily filtered geopotential height anomalies (e). It also shows, for the 66 WIEs, the spatial distribution of daily filtered rainfall anomalies (b), daily filtered OLR anomalies (d) and the daily filtered geopotential height anomalies (e). Dotted lines on all of these composite maps indicate regions where the values are significant at the 95% level. Line vectors indicate daily filtered wind anomalies at 850 hPa level composed over SIEs (column left) and WIEs (column right). It is clear that the impact of the 25–70 day ISO on precipitation strongly depends on the intra-seasonal events observed, the strong and the weak events. Figure 6a shows positive rainfall anomalies expanding mainly over most parts of the study area, all around latitudes 10°N–10°S and longitudes 10E–40°E; suggesting above-normal precipitation over these regions during SIEs. This spatial structure of intra-seasonal rainfall corroborates well with the work of Sandjon et al.^45^, who documented three dominant modes of intra-seasonal variability in CA, with spatial loadings centered over northern Congo, southern Ethiopia and southwestern Tanzania, respectively. In Fig. 6b, we can observe a dipolar structure in the spatial distribution of the filtered precipitation anomalies. This dipole is characterized by a strengthening of negative anomalies in the eastern part (15°S–5°N,25°E–40°E) and positive anomalies in the western part (15°S–5°N; 5°E–20°E) of our study area. This distribution of rainfall anomalies is not uniform all over the study region. There are points where the anomaly is almost zero and others where it is the highest. These two figures are similar to the structure of ISO described in Fig. 3, for EOF 1 during SIEs and EOF 2 during WIEs, respectively. The filtered OLR anomalies during SIEs (Fig. 6c) show negative values throughout the study area. This suggests that SIEs bring anomalous convection, with the core centered over eastern Central Africa and on the part of the Indian Ocean of the region. In Fig. 6d, is shown the distribution of both positive and negative filtered OLR anomalies throughout our area of interest. An East–West dipolar structure can be observed, with negative anomalies over the western Congo Basin and positive anomalies in the East. This matches well with the structure of rainfall anomalies in Fig. 6b, suggesting that this anomalous convection can be responsible for the rainfall pattern previously observed. The geopotential height is the height of the visible pressure surface. It is very essential for locating troughs and ridges which are the upper-level counterparts of surface cyclones and anticyclones (Gunta et al.^60^).

In Fig. 6e, negative filtered geopotential height anomalies at 925 hPa during SIEs are observed over the region, with maximum values centered around the north (10°N–15°N; 5°E–30°E) and over the Indian Ocean portion of the study area. This suggests a cold air temperature that can contribute to rainfall generation over these regions. During the WIEs, positive anomalies of filtered 925 hPa geopotential height (Fig. 6f) feature the northern part of the region along latitudes 5°N–15°N and the longitudes 10°E–35°E; and over the north of Zambia and a part south Congo Basin. These high geopotential height anomalies are associated with the anticyclonic circulation at 850 hPa level over the North hemisphere (clockwise) and the Southern hemisphere (counterclockwise). Negative anomalies are observed over the northeast of the region, whereas zero or near-zero anomalies characterize the rest of the study area. These positive anomalies would depict suppressed convection associated with vertical shrinking of the convection band that enhances cold air divergence in lower layers, therefore reducing precipitation (Gunta et al., 2022).

However, it is important to note that the dominant tropospheric circulation at 850 hPa during SIEs (Fig. 6a, c and e), is the low level westerly flows from the Equatorial Atlantic Ocean with a latitudinal extension between 5°S and 5°N, that penetrate the landmass and change southeasterly around the Congo Basin, then stop on the East African highland. And in the Eastern part, the dominant feature is the Northwesterly flow from the Indian Ocean that penetrates the continent through Tanzania and Nairobi East coast, and ends on their western coast. These westerly and Easterly flows bring cold air from the Atlantic and Indian Ocean to the continent and enhance precipitation. During WIEs the circulation features are the easterly flow from east Africa high lands to the Atlantic coast where it takes the southward direction, over the equatorial line. This feeds the convection observed over the Congo basin. We can also observe a divergent circulation over the eastern part (10°S to 10°N, 25°E to 40°E) which contributes to the drying of this region during the WIEs. Areas over which we have a divergent circulation correspond to areas of reduced rainfall (negative rainfall anomaly) while regions of convergent circulation correspond to increased rainfall (positive rainfall anomaly). This circulation pattern is responsible for the dipolar distribution of rainfall anomalies in the region during WIEs. For that matter, Longandjo and Rouault^61^, suggested that Congo basin cells may play an important role in rainfall redistribution over Central Africa.

**Figure 7:**
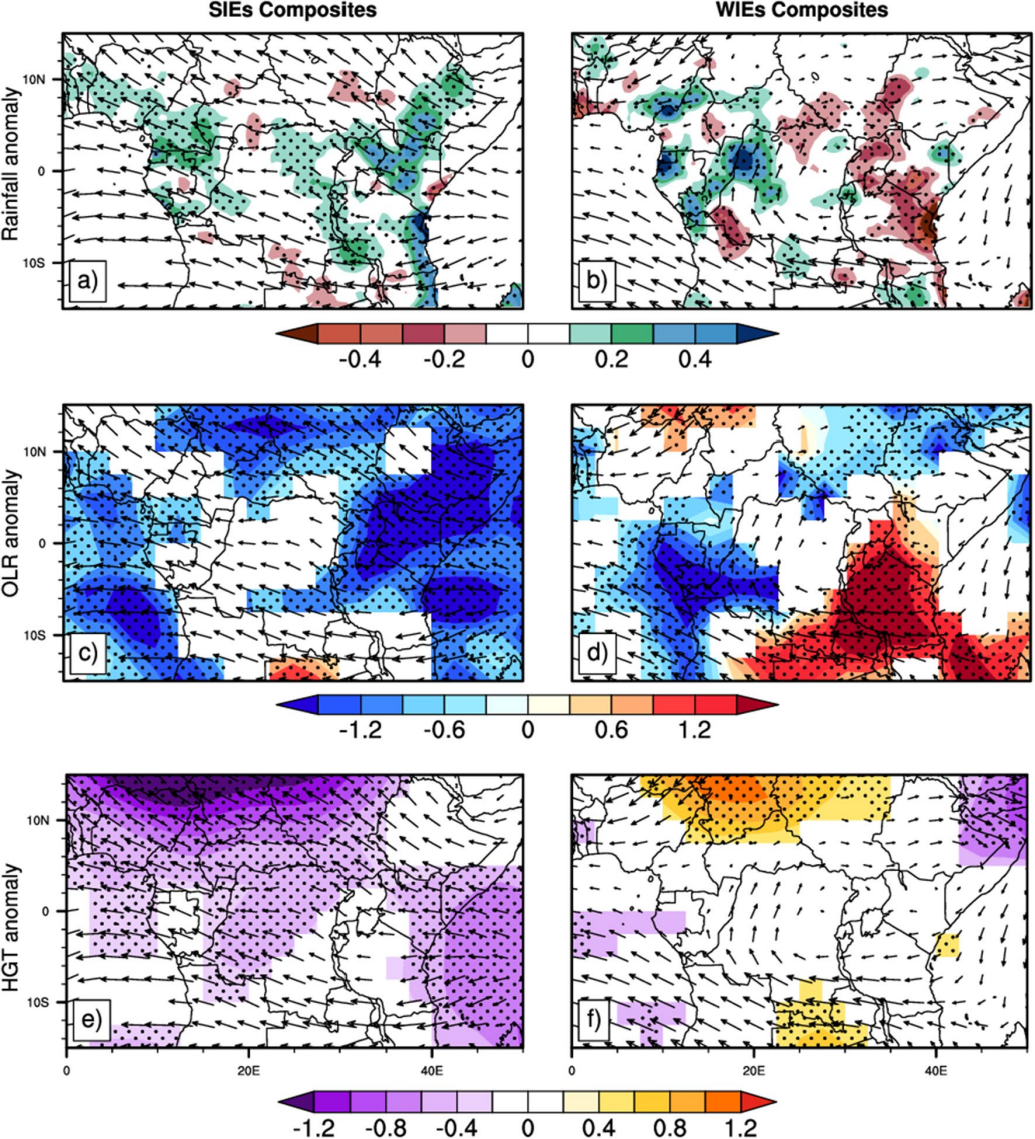
Same as in Fig. 3, but arrows indicate daily filtered wind anomalies at 200 hPa. Anomalies between − 0.01 and 0.01 m/s are not represented.

At 200 hPa, (Fig. 7) the dominant pattern of circulation during SIEs (Fig. 7a,c,e) is a strong easterly flow over the region from the Indian Ocean that crosses the continent, sloping northwestward above the latitude 5°N, southwestward below the latitude 10°S. This suggests very strong convective activity over the region. During WIEs (Fig. 7b,d,f), the dominant patterns at upper level are the southeasterly flow that crosses the continent over the southern part and falls in the Atlantic ocean; and the cyclonic circulation over East regions, from 20°E to 50°E and 5°S to 5°N. This circulation creates a subsidence zone extended over the longitude 20°E to 50°E (area that experiences reduced rainfall during WIEs). The wind anomaly patterns at 850 hPa and 200 hPa during SIEs were found to be conducive to the development of convection as shown by the enhanced negative OLR anomaly (Fig. 3c); and to the maintenance of cold air temperature (enhanced negative geopotential height anomalies in Fig. 6e), which would explain the positive rainfall anomalies over the region.

Studies of Farnsworth et al.^62^ revealed that the region Central Africa is very complex, with different mechanisms influencing rainfall in each region and season. It is therefore evident that circulation features at upper and lower layers during SIEs and WIEs are partly responsible for the differences in the spatial distribution of rainfall over Central Africa. Nevertheless, other mechanisms still need to be investigated in order to have more information about the association.

One main question raised here is, how much is the contribution of the intra-seasonal rainfall during the ISO, on the total seasonal precipitation of MAM? The ISO rainfall contribution in terms of percentages is calculated as follows:

7 $$\frac{{\underline{{P}_{iso}}}-{{\underline{{P}_{MAM}}}}}{\underline{{P}_{MAM}}}\times 100$$

where $\underline{{P}_{iso}}$ is the average over ISO (SIEs/WIEs) of the daily filtered rainfall anomalies. $\underline{{P}_{MAM}}$ is the total average over all days of MAM season of the filtered rainfall anomalies.

**Figure 8:**
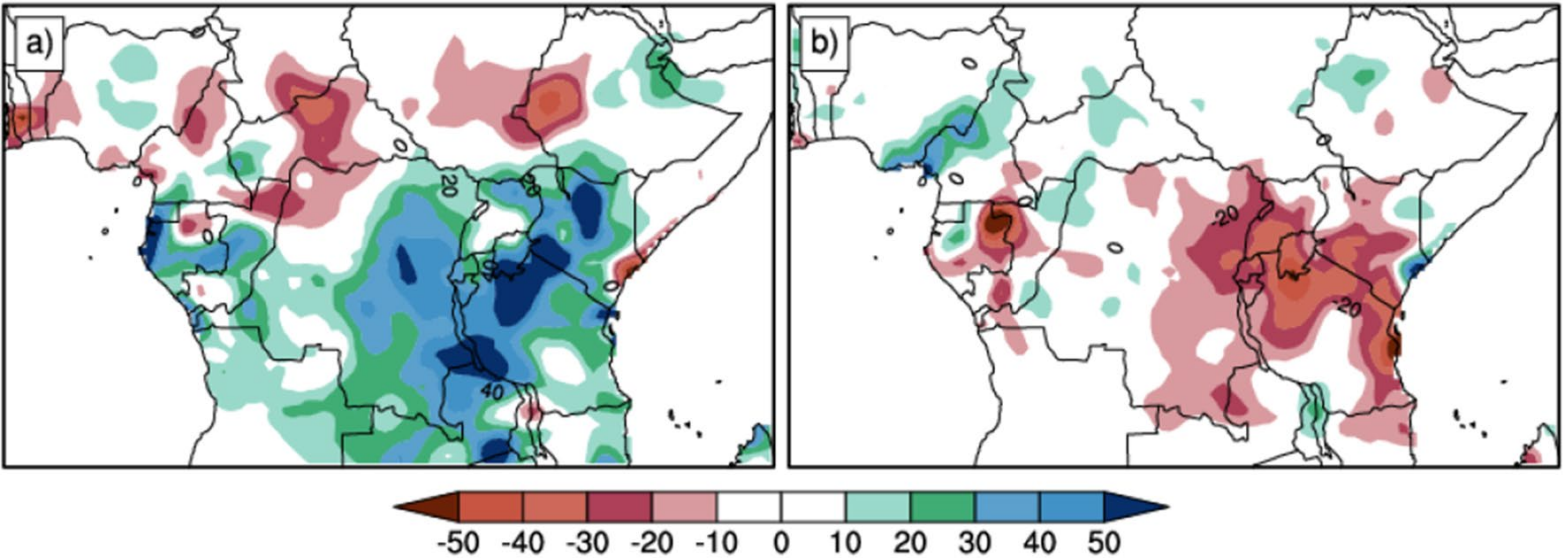
Spatial distribution of the impact rate for SIEs composite (**a**) and WIEs composite (**b**) in percentage.

Overall, the spatial rainfall anomaly pattern associated with the strong and weak IEs, described in section "Results and discussion".b, is reflected in the intra-seasonal rainfall impact. The percentage of contribution due to the SIEs (Fig. 8a) ranges from 20 to 30% over a large part of the region extending 5°N to 15°S of Latitude and 12°E to 40°E of Longitude; reaching around 50% of the daily rainfall anomaly over Lake Victoria, Northern Tanzania, Central Kenya and Lake Malawi. Conversely, during the weak IEs, values ranging around − 20% and − 50% are observed over the eastern part of the region (Fig. 8b), indicating a very weak contribution to total seasonal rainfall during WIEs.

### Relationship with MJO

We first evaluate MJO impacts on the intraseasonal variability of rainfall, convective activity and circulation in Central Africa during the 8 MJO phases^43^. It is clear that changes in the spatial distribution of intra-seasonal rainfall are from one phase of the MJO to another.

During the 40 rainy seasons of the analysis period (1980–2019) of this study, the number of days under each of the eight strong MJO Phases varies from 238 in Phase 5 to 342 in Phase 8 (Table 1).

**Table 1:** Number of MJO events per phase, during the MAM season for the whole study period (1980–2019).

Phases	Number of case
Phase 1	310
Phase 2	285
Phase 3	308
Phase 4	281
Phase 5	238
Phase 6	259
Phase 7	310
Phase 8	342
Total	2333
SIEs	71
WIEs	66

**Figure 9:**
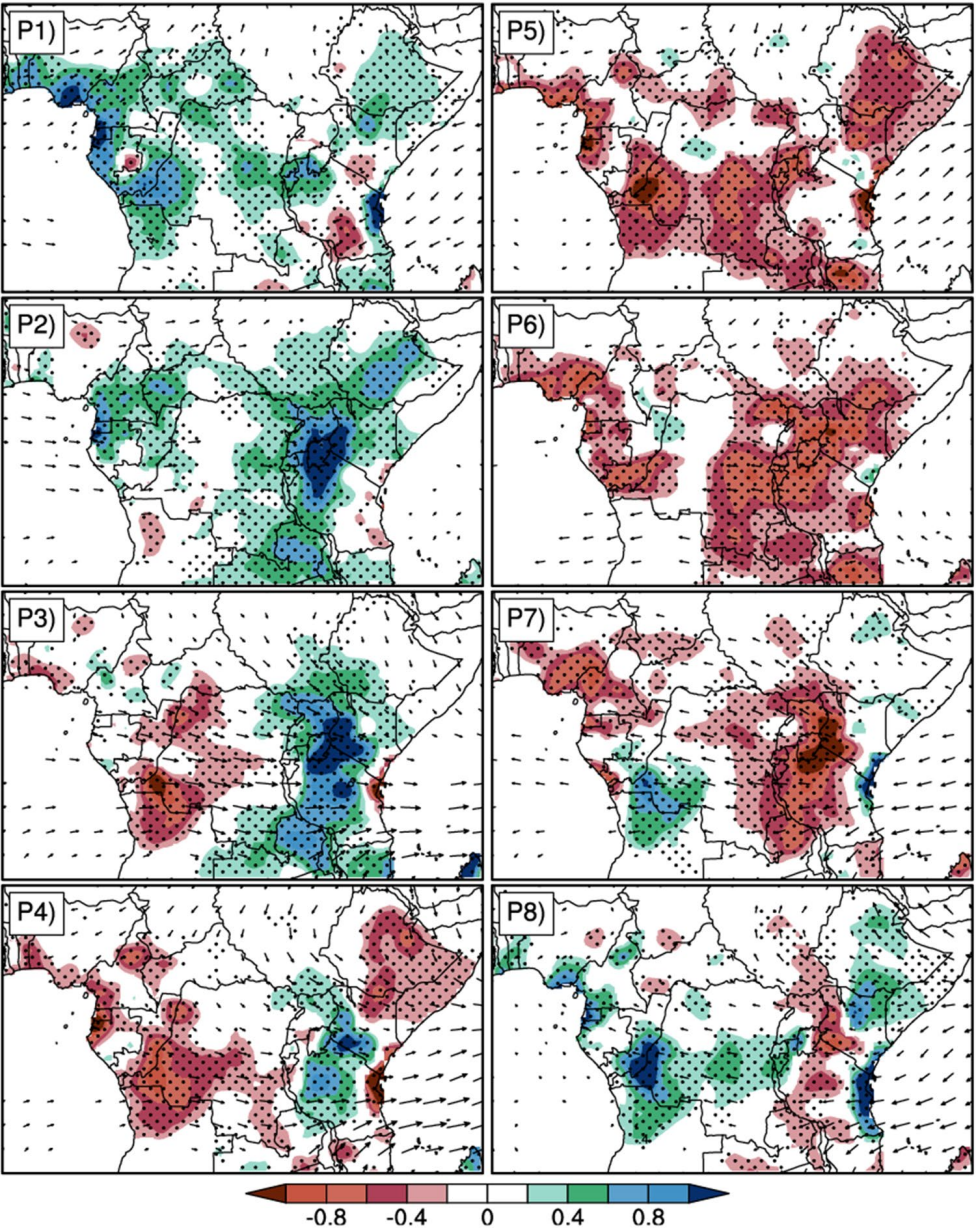
Composite of the daily filtered precipitation anomalies for each phase of the MJO. Anomalies between 0.05 mm and 0.05mm^2^ are not represented. Dotted lines indicate areas where the t-test is significant at 95%. Arrows indicate filtered wind anomalies at 850 hPa. Anomalies between − 0.01 and 0.01 m/s are not represented, and the reference magnitude of the wind is 2 m/s.

Figure 9 displays in color, the spatial loading of daily filtered rainfall anomalies composite over each phase of MJO according to the RMM index for MAM season. Arrows in each phase indicate the composite of daily filtered horizontal wind anomalies. During phase 1, the rainfall anomalies are positive over most parts of Central Africa (− 10°S, 10°N), indicating enhancement of precipitation. Few negative anomalies are observed over Southwest of Tanzania and South of Nairobi. During Phase 2, the intra-seasonal rainfall anomaly patterns were fewly similar to that of Phase 1 but for noticeable changes in the strength of the anomalies. One of those changes is that the region which experienced the maximum positive anomalies in Phase 1 have shifted Eastward from 0°–25°E to 25°E–40°E in Phase 2. With the appearance of maximum positive anomaly over North Tanzania, Rwanda and East of Kenya. These positive rainfall anomalies indicate an increase of rainfall over the regions. Phase 3 shows a dipolar-like pattern of the spatial distribution of rainfall anomalies over Central Africa. The Eastern part (− 10°S, 10°N; 0°, 25°E) experiences negative anomalies while the Western part (− 10°S, 10°N; 25°, 40°E) experiences positive anomalies. Compared to Phase 2, regions of positive anomalies in phase 3 have shifted Eastward with reduction of the intensity. While the negative anomalies tended to propagate Eastward and strengthen. In Phase 4, the spatial distribution of rainfall anomalies was almost the same as in Phase 3, but with weakened and reduced spatial extension of positive anomalies (over Central and western Tanzania, Southern and western Kenya, and Uganda) and strengthened and increased spatial extension of negative anomalies. However in the Eastern part (35°E–50°E) of the study area, the region was nearly divided into two halves of opposite rainfall anomalies with positive values over Southwestern part and negative values over the Northeastern and the Indian Coasts. Phases 5 and 6 show strong negative rainfall anomalies over most parts, which was nearly opposite to that during Phases 1 and 2. During the subsequent phase (Phase 7), the spatial distribution of rainfall anomalies is nearly opposite to that in Phase 3 (Wet regions in Phase 3 are dry in Phase 7 and inversely). This same observation is noticed in Phase 8 according to Phase 4. These results are nearly consistent with that of Omeny et al.^63^ which revealed strong association between East African rainfall and the MJO to the west of the region, especially around Lake Victoria.

It is clear that during its life cycle, MJO favored strengthening rainfall during the first two phases and weakening it in phases 5 and 6. This strengthening rainfall shifted Eastward in Phase 3 and in Phase 4 during which it tends to disappear, and started reappearing in Phases 7 and 8 in the Western parts. This finding is also in phase with that of Emily Hogan et al.^64^ who found that the MJO has a large impact on the intra-seasonal variability of precipitation in East Africa, with a strong dependence on its phases. They also suggested the existence of a dipole over the western highlands and the east coast that changes in sign over the lifetime of the MJO. Vashisht and Zaitchik^65^ mentioned that daily rainfall responses depend on MJO phases.

During the above phase 1, the circulation is too weak over the most area, excepted the southwesterly flow over the Indian Ocean which stops on the eastern coast; and the westerly wind anomalies that propagate over the Atlantic Ocean and ends at the continental coasts. The circulation in phase 2 is almost identical to phase 1, except that the westerly wind anomalies strengthened in speed and penetrated the continent around latitudes 5°S–5°N, up to the East African mountain ranges where they stopped. However, the easterly winds over the Indian Ocean weakened considerably. During phase 3 the west to east wind circulation strengthened considerably and flowed over the Indian Ocean from the Atlantic Ocean across the entire continental area. This low-level Westerly has shifted southward in the southern hemisphere and evolves between 5°S and 10°S. It progressively blows cold air Eastward over the land. During Phase 4, the circulation has considerably weakened over the western part (0°–30°E) and strengthened over the Eastern part (30°E to 50°E and 5°N to 10°S) mainly across the Indian Ocean. In the subsequent phase (phase 5), it has weakened over the entire area except across the Indian Ocean over which it is oriented Northeastward. Over the Western part (0° to 30°E), the weak circulation is Northeastward in the North hemisphere and Eastward across the equator and the lower latitude of the South hemisphere. In Phase 6 the Easterly flow that began in Phase 5 has now strengthened over the longitude 0° to 30°E and considerably weakened along the longitude 35°E to 50°E. The circulation observed from Phase 1 to the Phase 4 has changed oppositely respectively from Phase 5 to Phase 8.

The spatial distribution of the horizontal circulation in the lower layers is very impacted during the life cycle of the MJO, and seems to be phase dependent. It is associated with the Eastward movement of the positive rainfall anomalies distribution from Phase 1 to Phase 8.

**Figure 10:**
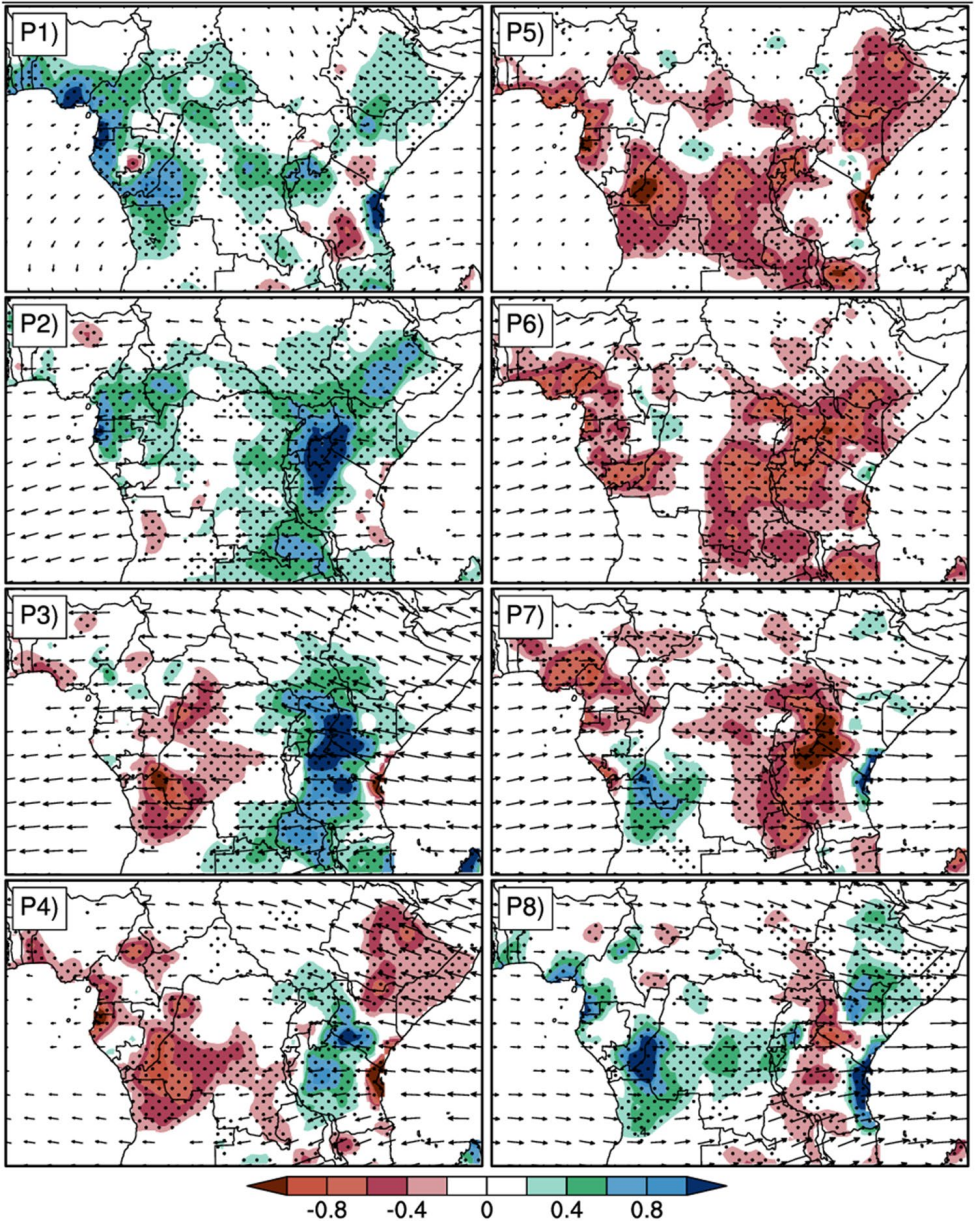
Same as in Fig. 9 but arrows indicate filtered wind anomalies at 200 hPa. Anomalies between − 0.01 and 0.01 m/s are not represented, and the reference magnitude of the wind is 5 m/s.

Figure 10 is the same as Fig. 9, but vectors represent the zonal filtered wind anomalies at 200 hPa. The dominant features of the circulation from Phase 1 to Phase 8 are a strong Easterly flow (phases 2, 3 and 4) that crosses the entire study area from the Indian Ocean to the Atlantic Ocean, and the strong Westerly flow (phases 6, 7 and 8) over the region. Meanwhile, Phase 1 shows a very weak circulation over almost all the land region, except Northern Ethiopia and the Indian Ocean that experienced a Westerly flow from the East coasts over the longitude 35°E to 50°E. The Atlantic Ocean is recovered by a north–south flow from the continental coasts. Phase 5 also shows very weak circulation over the continent. In contrast to Phase 1, the easterly winds flow over the Indian Ocean and stop around the coast, and the westerly winds over the Atlantic Ocean stop at the coast.

**Figure 11:**
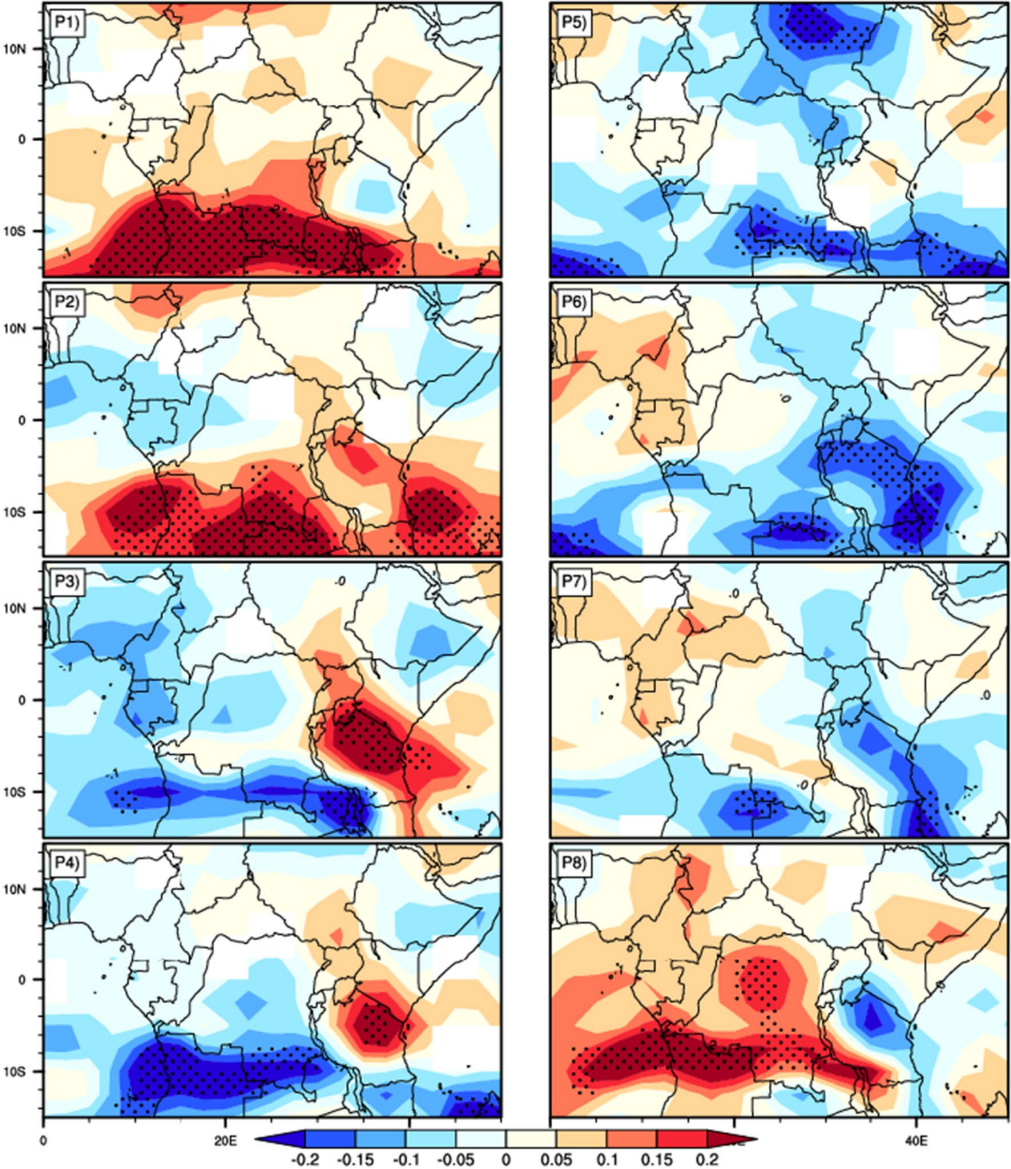
Longitude–Latitude diagram of MSE anomalies composited over the 8 phases of MJO in Central Africa. The diagram is plotted at 850 hPa. Dotted indicates areas where the t-test is significant at 95%. For each phase, the number of the phase is indicated at top left of the plot.

In the above paragraph, changes in the rainfall anomaly patterns associated with the eight Phases of MJO were examined. Now we explore how these changes are related to the changes in convection over the Central Africa region, using the moist static energy (MSE) anomaly. Figure 11 shows the composite of daily MSE anomalies for the different Phases of the MJO. It can be seen that the effect of MJO on MSE is also very phase dependent. During phase 1, positive MSE anomalies cover the entire region, with highest and significant values over the southern part, extending from 5°S to 15°S of latitude and 5°E to 35°E of longitude. In phase 2, these peak of positive anomalies has extended Eastward to the longitude 50°E, and some negatives anomalies started appear from the west coast of the Atlantic ocean above Equator, over Congo Brazzaville, Gabon, the southern Cameroon, the west of Congo Basin and the eastern part of the horn of Africa. In phase 3, the spatial coverage of the significant positive MSE anomalies have considerably reduced and are centered over most part of Tanzania and the southern part of Kenya. We can also observe a North–South gradient pattern of positive and negative MSE anomalies (positive anomalies over the North and negative over Southward) over the eastern part of the region, from 30°E to 45°E and 15°S to the Equator. In phase 4, negative MSE anomalies are also dominant over the region, as in phase 3. The significant positive anomalies are located only over the North of Tanzania, while the negative has extended over the southern part of the region, from 5°E to 50°E under the latitude 5°S. In phase 5 the entire region is dominated by negative MSE anomalies, with significant values over Sudan, a part of southern Congo Basin, Northern Zambia and over the southern regions of Atlantic and Indian oceans. The distribution of anomalies seem to be opposite to that in phase 1. During phase 6, the areas covered by negative anomalies start to shrink and we observe the formation of positive anomalies in the west of the study area, above 6°S latitude. The spatial distribution of these anomalies is the reverse of that of phase 2, in that the areas that were dominated by positive/negative anomalies in phase 2 are occupied by negative/positive anomalies in phase 6. The progression of these positive anomalies eastward will continue in phase 7 with a suppression of negative anomalies. The spatial pattern is opposed to that of Phase 3. In phase 8, the positive anomalies spread over almost the whole study area. The spatial distribution is almost the opposite of what we had in phase 4. As a result, areas with negative anomalies are extremely stable, traducing absence of convective activity and thus dry conditions. These areas match well those of Fig. 9 that experience reduced rainfall. However, areas with positive anomalies of MSE are less stable, favoring much convective activity and thus more wet conditions. These areas of above normal MSE at the surface also match well with those in Fig. 9 that experience positive rainfall anomalies. This result is similar to that of Yang et al.^66^ in their study of the annual cycle of East African precipitation.

The above-normal convection (characterized by positive anomalies of MSE at surface) that propagates eastward during phases 2, 3, and 4 of the MJO is accompanied by the eastward horizontal geostrophic wind flow in the upper layers. At the same time, the positive anomalies that appear and evolve eastward during phases 5, 6, 7, and 8 of the MJO are accompanied by the westward horizontal geostrophic wind flow in the upper layers. The horizontal westerly wind flows in the lower layers, carries cold air from the Atlantic Ocean in Pases 1, 2, 3 and 4, and distributes it over the continent in each region according to the topography. In Phases 5, 6, 7 and 8 the warm air from the Indian ocean is carried to the continent by the easterly flow that dominates in those phases.

To measure the similarity between the composite of filtered rainfall anomalies during SIEs(WIEs) and the composite rainfall during each of the MJO phases, we present in Table 2 below the uncentered pattern correlation between composited filtered rainfall anomalies during the MJO and ISO. There is a strong positive correlation (0.5) between MJO phases 1 and 2 and SIE. This suggests that the spatial distribution of the intra-seasonal rainfall over Central Africa during Strong ISO and the phases 1 and 2 of the MJO are nearly similar. Similarly, we observe a strong negative correlation (− 0.6) between MJO phase 6 and SIEs. This also suggests that the regions where an increase in rainfall is observed during phase 6 correspond to the regions where a suppression or a significant decrease in rainfall is recorded during SIEs. However, the correlation with the other phases is very weak (below 0.5), indicating that there is no similarity between these phases of the MJO and SIEs. The correlation between MJO phases and WIEs is very low overall, reaching 0.03 between phase 2 and WIEs; with the exception of the correlation between phase 7 and WIEs which substantially reaches 0.5. This means that there is a very weack similarity between the phases of the MJO and WIEs, except with phase 7 which bears a considerable resemblance.

**Table 2:** Uncentered pattern correlation between composite of filtered anomalies of precipitation according to each MJO phase and SIEs(WIEs).

	Phase 1	Phase 2	Phase 3	Phase 4	Phase 5	Phase 6	Phase 7	Phase 8
SIEs	**0.5**	**0.5**	0.3	− 0.2	− 0.4	**− 0.6**	− 0.4	0.3
WIEs	0.05	− 0.003	**− 0.3**	− 0.24	− 0.02	0.05	**0.4**	0.21

Significant values are in [bold].

## Summary and conclusion

In this study, we investigated the influence of the 25–70-day intra-seasonal variability on rainfall during the MAM season. Firstly, we defined strong and weak intra-seasonal events using the first two principal components (PC1 and PC2), derived from the EOF analyses of daily filtered anomalies of OLR data, from 1980 to 2019. This led to the identification of 71 SIEs and 66 WIEs over the entire study period. The life cycle of the ISO evnts is around 40 days (Fig. 4). The composite of daily filtered rainfall anomalies showed that during strong intra-seasonal events, the spatial distribution of rainfall is affected, giving rise to zones of above-normal rainfall. During weak intra-seasonal events, the spatial structure of rainfall is an East–West dipole like pattern, with suppressed rainfall over the East and enhancement rainfall in the West. The earlier (t0-20) and the last stages (t0 + 20) are bounded to reduced rainfall over most part of CA with pics within longitude 15°E to 50°E; while the intermediate stages (t0-10, t0 and t0 + 10) are related to an increase in precipitation over CA. During WIEs, the first stage (t0-20) is associated with increased rainfall over the Congo Basin and the East of East-African highland which extends and strengthens further at t0-10 over most part of the region. This spatial distribution is strongly modulated by the low level circulation and the geopotential height at intra-seasonal time scale, during the both SIEs and WIEs.

Results also revealed that the influence of MJO on the intra-seasonal rainfall clearly varies from one phase to another. Phases 1 and 2 generate heavy rainfall over most of the region; while during phases 3, 4, 7 and 8 there is an east–west dipolar distribution of positive and negative rainfall anomalies. During phases 5 and 6, we have a pronounced suppression of rainfall over the entire region. This influence is modulated by the low level circulation that brings cold (or warm) air from the surrounding Oceans to the continent.

The uncentered pattern correlation analysis revealed a similarity in spatial distribution between intra-seasonal rainfall during the SIEs and phases 1 and 2 of the MJO. As well, the correlation between rainfall anomalies during WIEs and MJO phase 7 also suggested a relationship of similarity in the spatial distribution of rainfall.

In sum, in this study, we showed that intra-seasonal oscillation (ISO) affects the spatial distribution of rainfall over Central Africa. Changes in the atmospheric circulation at low-level and high altitudes can be associated to those in the spatial distribution of rainfall in the region. The influence differs depending on the ISO event. Likewise, the MJO modulates rainfall in CA by generating a progressive eastward shift of the positive rainfall anomalies, from phase 1 to phase 8. Further inquiries are needed to better characterize the mechanisms regulating the interactions between ISO events and the MJO phases.

## Data Availability

The datasets analyzed during the current study are all available online: The OLR is available on the NOAA repository at https://www.ncei.noaa.gov/data/outgoing-longwave-radiation-daily/access/ The CPC reanalysis, developed by the NCEP/Climate Prediction Center, is available at https://psl.noaa.gov/data/gridded/data.cpc.globalprecip.html The NCEP-DOE Reanalysis 2 data is available from PSL at https://psl.noaa.gov/data/gridded/data.ncep.reanalysis2.html
